# Experimental Analysis and Mathematical Modeling on Mg-Li Alloy Sheets with Three Crystal Structures during Cold Rolling and Heat Treatment

**DOI:** 10.3390/ma10101167

**Published:** 2017-10-12

**Authors:** Yan Tang, Qichi Le, Tong Wang, Xingrui Chen

**Affiliations:** Key Lab of Electromagnetic Processing of Materials, Ministry of Education, Northeastern University, Shenyang 110819, China; tangyan361@163.com (Y.T.); wangtong@epm.neu.edu.cn (T.W.); 13390133268@163.com (X.C.)

**Keywords:** microstructural evolution, mechanical properties, mathematical relationship

## Abstract

The microstructural evolution, mechanical properties, and mathematical relationship of an α, α + β, and β phase Mg-Li alloy during the cold rolling and annealing process were investigated. The results showed that the increased Li element gradually transformed the Mg matrix structure from hcp to bcc. Simultaneously, the alloy plasticity was improved remarkably during cold rolling. In the annealing process, a sort of abnormal grain growth was found in Mg-11Li-3Al-2Zn-0.2Y, but was not detected in Mg-5Li-3Al-2Zn-0.2Y and Mg-8Li-3Al-2Zn-0.2Y. Moreover, the mechanical properties of alloy were evidently improved through a kind of solid solution in the β matrix. To accurately quantify this strengthening effect, the method of mathematical modeling was used to determine the relationship between strength and multiple factors.

## 1. Introduction

The Mg-Li alloy possesses many impressive advantages, such as a low density, high specific elastic modulus, high specific strength, good electromagnetic shielding, and damping property [1,2,3,4]. Therefore, it has been widely applied in the fields of weapon, automobile, aerospace, aviation, electronics, and military industries, etc. [5,6,7]. It has been reported that the addition of an Li element could change the Mg crystal structure by reducing the c/a ratio of the hexagonal lattice [8]. When the Li content is less than 5.7%, the alloy matrix structure will reveal the hexagonal close-packed (hcp) lattice [9]. When the Li content ranges from 5.7–11.3%, the hexagonal close-packed (hcp) structure of Mg will be transformed into a hexagonal close-packed (hcp)+body-centered cubic (bcc) dual-matrix [10]. When the Li content is more than 11.3%, the single body-centered cubic (bcc) structure will be presented wholly in the alloy [11]. Nevertheless, with an increasing Li content, the mechanical properties of the alloy, corrosion resistance, and high temperature resistance will decline [12]. These drawbacks will restrain its wide application in the national economy. Many researchers have found that studies should focus on its strength improvement. Hence, previous researchers have adopted many positive approaches to improve the strength of the Mg-Li alloy and have gained some good results [13,14,15,16,17]. Their methods involved alloying element addition (Al, Zn, Ca, Sr, and so on), rare earth element addition (Ce, Y, Nd, La, and so on), ageing and solid solution processes, as well as equal channel angular pressing, etc. However, there are few reports about cold rolling and annealing studies on an Mg-Li alloy sheet. Moreover, quantitative analyses and mathematical relationship modeling on the Mg-Li alloy have been rarely reported.

In this paper, the microstructural evolution, mechanical properties, and mathematical relationship of an α, α + β, and β phase Mg-Li alloy during the cold rolling and annealing process were investigated. 

## 2. Experiments

**Figure 1:**
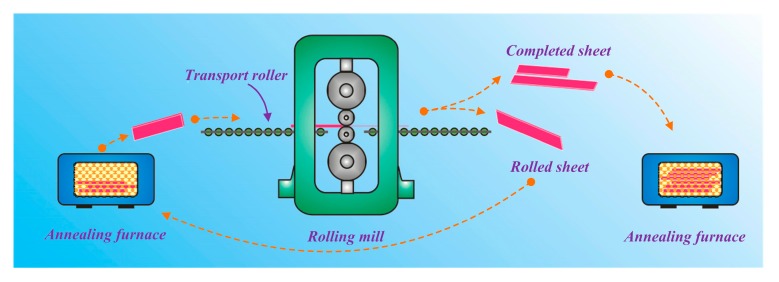
Rolling process diagram of the Mg-Li alloy sheet.

The Mg-Li alloys in this investigation involved Mg-5Li-3Al-2Zn-0.2Y, Mg-8Li-3Al-2Zn-0.2Y, and Mg-11Li-3Al-2Zn-0.2Y. The experimental materials were commercial pure Mg ingot, pure Li ingot, pure Al ingot, pure Zn ingot, and Mg-25%Y master alloy ingot. The ingots were melted in an iron crucible under the atmosphere of SF_6_, and simultaneously the flux mixture was used to keep the melt away from the air. Then, the melt was poured into the permanent mould to gain an as-cast alloy. The received cast alloys were then rolled into the sheets during the multi-pass process. After each pass rolling, the sheet was heated at 523 K for 15 min and then rolled in the next pass. The rolling reduction was set as 3%. In the end, a sheet with a thickness of 2 mm was obtained. The completed sheets were heat-treated at different temperatures for 24 h (473 K–573 K) and were then quenched into the cold water. The rolling flow chart of the Mg-Li alloy sheet is shown in Figure 1.

Metallographic specimens were polished mechanically and etched with a solution of 5 vol. % nitric acid alcohol. The microstructural observation was examined by an optical microscope (OM) and a scanning electron microscope (SEM, Oxford Instruments (China), Shanghai, China) equipped with an Oxford energy dispersive spectroscope (EDS, Oxford Instruments (China), Shanghai, China). The phase identification was performed with X-ray diffraction (XRD, PANalytical B.V. (China), Beijing, China). The mechanical properties of the alloys were investigated at room temperature on the universal testing machine with a strain rate of 1.0 × 10^−3^ s^−1^.

## 3. Results and Discussion

### 3.1. Microstructural Observation

**Figure 2:**
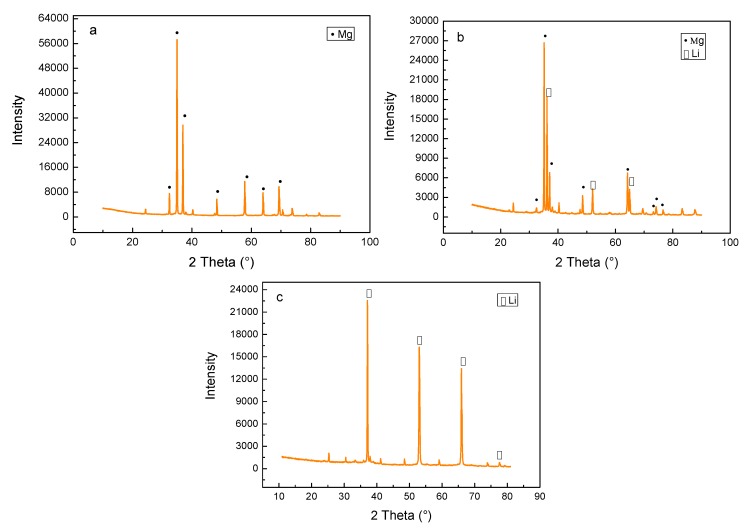
XRD patterns of as-cast alloys: (**a**) Mg-5Li-3Al-2Zn-0.2Y; (**b**) Mg-8Li-3Al-2Zn-0.2Y; (**c**) Mg-11Li-3Al-2Zn-0.2Y.

**Figure 3:**
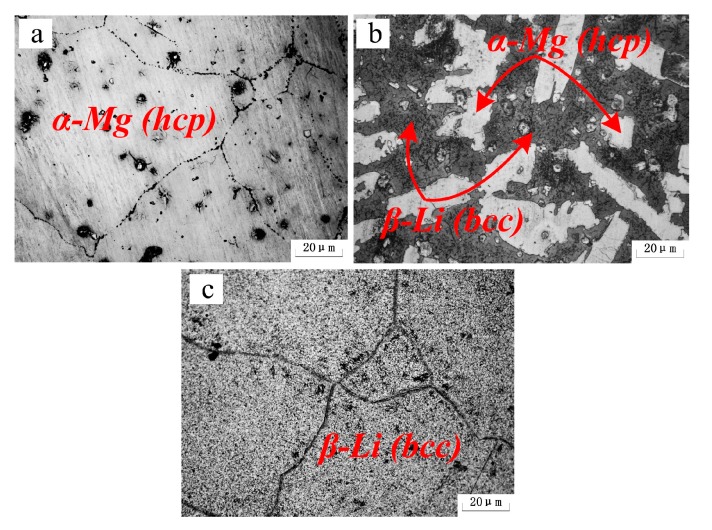
Microstructures of as-cast alloys: (**a**) Mg-5Li-3Al-2Zn-0.2Y; (**b**) Mg-8Li-3Al-2Zn-0.2Y; (**c**) Mg-11Li-3Al-2Zn-0.2Y.

Figure 2 shows the XRD patterns of the as-cast alloys. It indicates that a transformation between the lattice structures took place with increasing Li content. When the Li content was increased to 5%, the Mg-5Li-3Al-2Zn-0.2Y alloy was mainly composed of α-Mg (hcp) phase, as shown in Figure 2a. However, when the Li addition increased to 8%, some Li matrix peaks with relatively high intensity emerged, implying that the Mg-8Li-3Al-2Zn-0.2Y alloy was mainly composed of α-Mg+β-Li dual-phase, as shown in Figure 2b. When the Li content was further increased to 11%, the previous α-Mg peak was mostly replaced by the β-Li peak, deducing that the Mg-11Li-3Al-2Zn-0.2Y alloy primarily consisted of β-Li phase, as shown in Figure 2c. Figure 3 shows the microstructures of the as-cast alloys. Based on the previous XRD patterns, the microstructural evolution confirmed that the increasing Li element transformed the Mg lattice structure from hcp to bcc.

**Figure 4:**
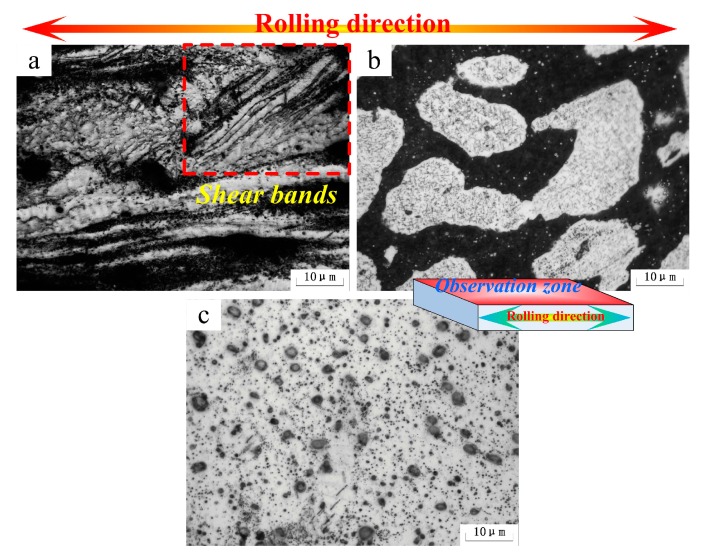
Microstructure of as-rolled alloys: (**a**) Mg-5Li-3Al-2Zn-0.2Y; (**b**) Mg-8Li-3Al-2Zn-0.2Y; (**c**) Mg-11Li-3Al-2Zn-0.2Y.

Figure 4 shows the microstructures of the as-rolled alloys. In Mg-5Li-3Al-2Zn-0.2Y (see Figure 4a), the α-Mg (white zone) and mixed secondary (dark gray zone) phases were both elongated and well distributed along the rolling direction. Moreover, some shear bands aligned with the rolling direction were presented in the α-Mg matrix, indicating that the α-Mg alloy revealed the poor ability of plastic deformation at low temperature. This shear deformation resulted from the incomplete slip between the crystal lattices during the deformation process. The basal slip was the predominant slip mode during the early deformation. However, the normal slip deformation could not continue after basal slip finishing as the non-basal slips were difficult to activate at low temperature. Hence, the shear deformation emerged and then took over the primary deformation mode in the later deformation process.

In Mg-8Li-3Al-2Zn-0.2Y (see Figure 4b), the β-Li phase (black zone) was obviously elongated along the rolling direction and shear bands were not found. It indicated that the plasticity of the β-Li matrix was much better than that of the α-Mg matrix. The difference between them was ascribed to the characteristics of both crystal structures. The β-Li with the bcc structure possessed many more slip systems and a symmetrical crystal structure compared with α-Mg. Hence, the coordinating deformation and dislocation movements between grains in the β-Li matrix are an advantage compared to the α-Mg matrix. Throughout the rolling process, no shear deformation could be observed in the α-Mg phase. It illustrated that the increasing Li element gradually improved the plasticity of the α-Mg matrix. The reason for this improvement was that the Li addition effectively reduced the parameter c/a axial ratio of the Mg crystal structure, promoting the non-basal slips, such as {10-10} prismatic and {10-12} pyramidal slips [2]. Thereby, the deformation of the α-Mg matrix would be relatively amenable in the rolling process. In Mg-11Li-3Al-2Zn-0.2Y (see Figure 4c), the markedly elongated β-Li microstructure was well distributed along the rolling direction. The results confirmed that the β-Li phase possessed a relatively outstanding plasticity.

**Figure 5:**
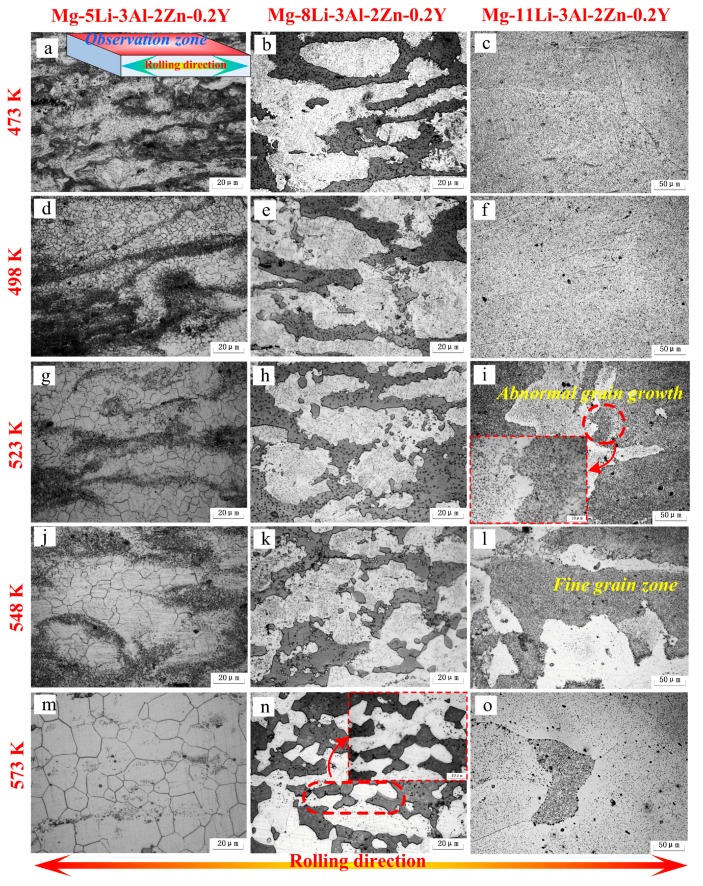
Microstructures of the as-rolled alloys annealed at different temperatures for 24 h.

Figure 5 shows the microstructural evolution of the as-rolled alloys during annealing at different temperatures for 24 h (473 K–573 K). In Mg-5Li-3Al-2Zn-0.2Y, a small number of fine equiaxed grains at 473 K were presented in the matrix, indicating that static recrystallization behavior occurred at approximately 473 K. As indicated in Figure 5a, the deformed microstructure still occupied the most area in the matrix. At 498 K, a great number of small equiaxed grains emerged from the matrix and the mean grain size was measured as 3.1 μm. Additionally, the previous deformed microstructure was apparently substituted by the recrystallized microstructure, illustrating that the recrystallization behavior had taken place, as shown in Figure 5d. With the annealing temperature increasing to 523 K, the grain growth phenomenon was gradually manifested, in which the mean grain size was measured as 8.9 μm, as shown in Figure 5g. In the range of 548 K–573 K (see Figure 5j,m), the coarse grains and clear grain boundaries emerged, wherein the mean grain size at 548 K and 573 K was 13.5 μm and 20.3 μm, respectively.

Considering the recrystallization and grain growth, the grain (nuclei) growth speed could be characterized by Equation (1). As indicated in Equation (1), the grain (nuclei) growth speed was directly proportional to deformation energy. It illustrated that serious deformation would contribute to accelerating grain growth at the same condition, which could be explained by the fact that the deformed zone with high energy would provide a fast channel for the atomic diffusion to promote highly frequent nucleation. Equation (1) could be further simplified as Equation (2). From the Equation (2), the decreasing growth activation energy would accelerate the grain (nuclei) growth, which could be explained by the fact that the relatively high dislocation density could reduce barriers over which the atoms would stride in order to diffuse thoroughly. Hence, the relatively low activation energy would be in favor of grain growth. The mechanisms of recrystallization and grain growth behaviors should be attributed to the transition process from high free energy to low free energy.

(1) $$\left\{ \begin{array}{l} V=\frac{D_{B}}{KT}\cdot \frac{E_{S}}{\lambda }\\ D_{B}=D_{0}\exp(-\frac{Q_{g}}{RT}) \end{array}\right.$$

where V is the grain (nuclei) growth speed, $D_{B}$ is the diffusion coefficient at the grain boundary, λ is the interface width, K is a constant, $E_{S}$ is the deformation energy, R is the gas constant, and T is the temperature.

(2) $$V=V_{0}\exp(-\frac{Q_{g}}{RT})$$

where $Q_{g}$ is the growth activation energy. 

In Mg-8Li-3Al-2Zn-0.2Y, in the annealing period of 473 K–498 K, the two matrices did not generate clear microstructural evolution, wherein the elongated α and β phases were still distributed along the rolling direction, as shown in Figure 5b,e. However, a little variation was gradually exhibited around the β phase edge where the small β grains with a granule shape emerged at 523 K, as shown in Figure 5h. It indicated that the recrystallization behavior in the β matrix might take place at approximately 523 K. With the temperature increasing to 548 K, the recrystallized β grains became more numerous than before, as shown in Figure 5k. When the alloy was annealed at 573 K, the mean grain size of β was measured as 15.6 μm, as shown in Figure 5n. The whole recrystallization process in the β matrix should be ascribed to a recrystallization mechanism where the combination between the subgrains and dislocation absorbing was the main evolution process. In addition, the above–mentioned combination resulted from the transformation among the grain boundary category from a low angle to high angle [18]. 

In Mg-11Li-3Al-2Zn-0.2Y, no obvious microstructural variation could be found below 498 K, as shown in Figure 5c,f. When annealing treatment was set at 523 K (see Figure 5i), a certain number of tremendous grains emerged around the fine grain zone, which were almost 100 times as big as the fine grains. With the increased annealing temperature (548 K), this phenomenon became more apparent, as shown in Figure 5l. With annealing at 573 K, the coarse grains were predominant, and simultaneously a small number of residual fine grains were presented in the matrix, as shown in Figure 5o. This behavior mentioned above belonged to the abnormal grain growth whose activation could be characterized by the driving force during the abnormal grain growth (see Equation (3)). As indicated in Equation (3), the activation of abnormal grain growth was the result of an interfacial energy difference between grains. Furthermore, the grain size in the matrix was closely associated with the interfacial energy. Obviously, the size of advantageous grain was coarser than that of fine grain. Hence, *p* > 0 and the abnormal grain growth would be activated.

(3) $$\left\{ \begin{array}{l} p=\Delta \gamma \\ \Delta \gamma =a\gamma (\frac{1}{\bar{D}}-\frac{1}{D}) \end{array}\right.$$

where p the is driving force, Δγ is the interfacial energy difference, $\bar{D}$ is the mean grain diameter of fine grain, D is the grain diameter of advantageous grain, and a is a constant.

In the stable recrystallized matrix, a small number of advantageous grains were treated as the nuclei for abnormal grain growth. These advantageous grains could grow preferentially because of the inconsistently dissolved secondary phases. Moreover, their morphology category almost belonged to the polyhedron (>6) whose surface was revealed as concave in favor of grain boundary diffusion. In the growing process, the existence of a particular orientation difference contributed to enhancing the migration velocity to effectively promote the abnormal grain growth. 

**Figure 6:**
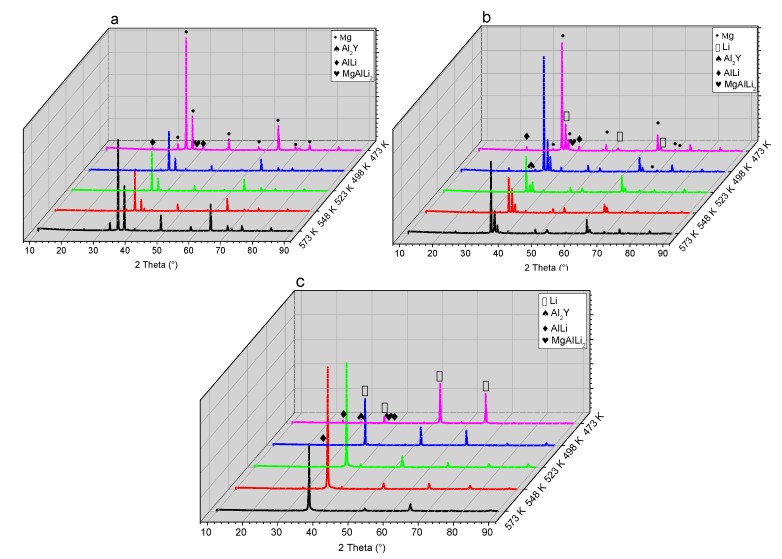
XRD patterns of as-rolled alloys annealed at different temperatures for 24 h: (**a**) Mg-5Li-3Al-2Zn-0.2Y; (**b**) Mg-8Li-3Al-2Zn-0.2Y; (**c**) Mg-11Li-3Al-2Zn-0.2Y.

Figure 6 shows the XRD patterns of the as-rolled alloys during annealing for 24 h (473 K–573 K). In Mg-5Li-3Al-2Zn-0.2Y (see Figure 6a), with increasing temperature, no evident phase evolution could be observed. In Mg-8Li-3Al-2Zn-0.2Y (see Figure 6b), the peak altitude of AlLi phase manifested a little fluctuation with increasing temperature. However, the other phases remained stable. In Mg-11Li-3Al-2Zn-0.2Y (see Figure 6c), the AlLi peak variation was consistent with that in Mg-8Li-3Al-2Zn-0.2Y, indicating that the elevating temperature changed the peak of AlLi phase. The above-mentioned results illustrated that the annealing temperature might influence the AlLi phase solubility in the matrix.

**Figure 7:**
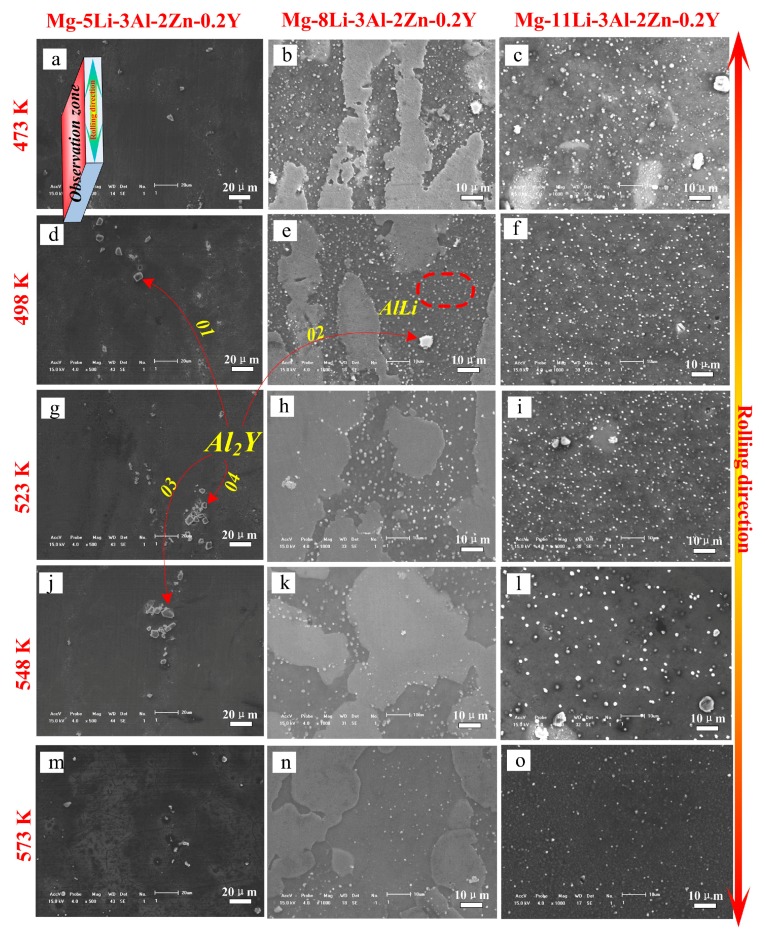
SEM images of the as-rolled alloys annealed at different temperatures for 24 h.

**Figure 8:**
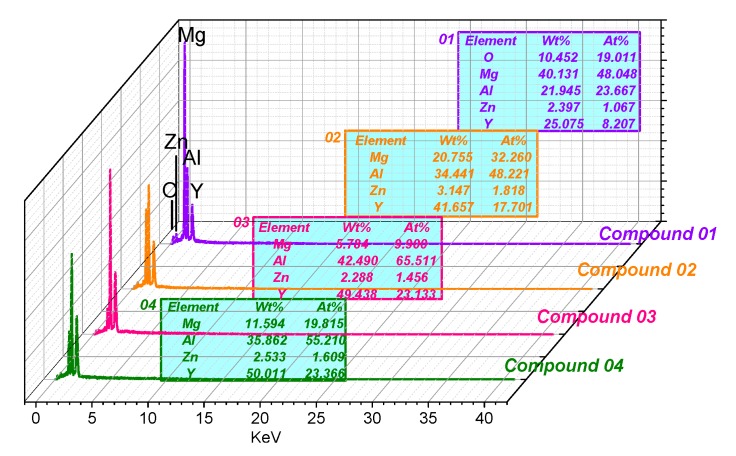
EDS results of Al_2_Y phase marked in Figure 7.

**Figure 9:**
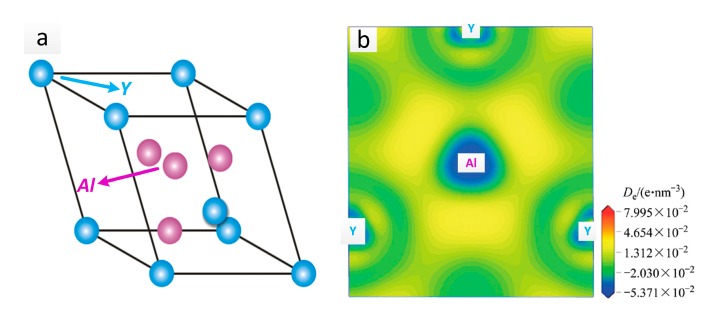
Al_2_Y crystal structure (**a**) and its electronic density difference (De) of the (111) plane (**b**).

Figure 7 shows the SEM results of the as-rolled alloys during annealing for 24 h (473 K–573 K). In Mg-5Li-3Al-2Zn-0.2Y, the crushed Al_2_Y phase was steadily distributed in the matrix with increasing temperature. Meanwhile, no other obvious change was observed in the matrix, as shown in Figure 7a,d,g,j,m. The EDS results were obtained to make a better analysis for Al_2_Y phase, as shown in Figure 8. The Al_2_Y stability that would not be affected by the annealing temperature should be attributed to its special crystal structure. The Al_2_Y crystal structure and its electronic density difference (De) of the (111) plane are shown in Figure 9a,b [19]. The stability mechanism resulted from an intense interaction between the valence electron orbits of the Al and Y atoms, in which a sort of Laves phase structure was made. Additionally, the metallic, covalent, and ionic bonds involved in the Al_2_Y lattice structure also played a very important role. The crystal structure parameters of Al_2_Y are listed in Table 1 [19].

In Mg-8Li-3Al-2Zn-0.2Y, a great many dispersed white granules were well distributed in the β matrix at 473 K (see Figure 7b). With annealing at 498 K, these dispersed granules became less numerous. In the annealing period of 523–548 K (see Figure 7h,k), the number of white granules apparently decreased, implying that they decomposed and dissolved into the β matrix. According to the previous XRD analysis, these dissolved granules were considered as AlLi phase. At 573 K (see Figure 7n), a small amount of residue was only maintained in the microstructure. Furthermore, a similar dissolution law occurred in the Mg-11Li-3Al-2Zn-0.2Y alloy, as shown in Figure 7c,f,i,l,o.

**Figure 10:**
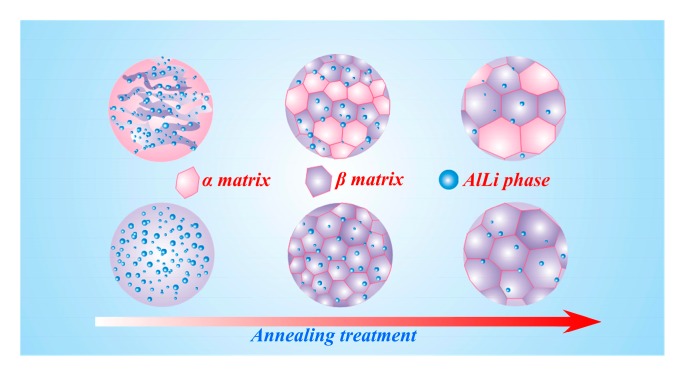
Flow chart of solid solution of the Mg-Li alloy.

The atomic solid solubility in the matrix could be analyzed by the Hume-Rothery empirical rule, which was named the “15%” rule (see Equation (4)). As indicated in Equation (4), when *δ* is more than 15%, the solubility of the dissolved atom in the matrix is extremely low because the relatively big radius difference limits the atomic dissolution, promoting the formation of an intermetallic compound. According to the calculation, *δ* on Li-Al was only 5.9%. Thereby, Al solubility in the β matrix was relatively high. In addition, the bonding energy between the Al and Li atoms in the intermetallic compound was relatively low. Therefore, based on the above-mentioned analysis, the increased temperature would promote the decomposition and dissolution of AlLi phase during the annealing process. The solid solution law with the annealing process is shown in Figure 10. It described the microstructural evolution law in the α and β matrices. In this evolution, the recrystallization phenomenon gradually emerged in the two matrices during annealing treatment. Meanwhile, the amount of AlLi phase in the β matrix decreased gradually, indicating that the solid solution behavior in the β matrix could be gradually activated by an increasing temperature. The recrystallization behavior should be ascribed to the dislocation density reduction and grain boundary migration. In addition, the increasing temperature accelerated the atomic diffusion and promoted the solid solution process.

(4) $$\delta =(\frac{\left|D_{a}-D_{b}\right|}{D_{a}})\times 100\%>15\%$$

where $D_{a}$ is the matrix atom diameter and $D_{b}$ is the dissolved atom diameter.

### 3.2. Mechanical Properties

**Figure 11:**
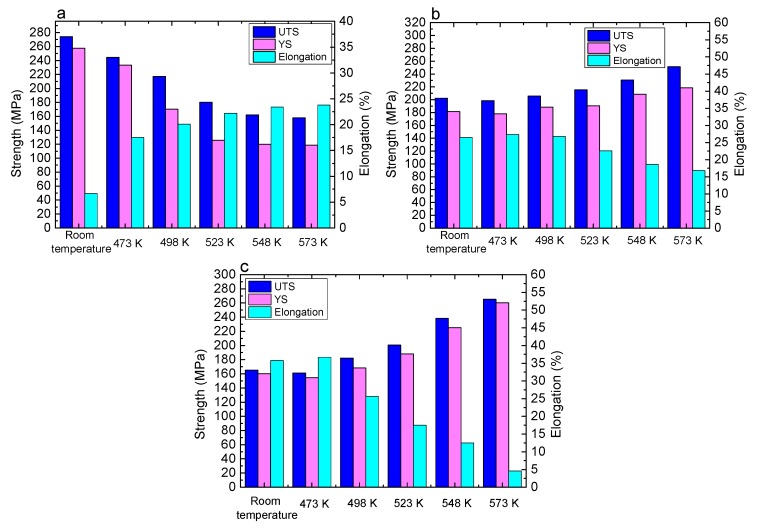
Mechanical properties of three alloys at different conditions: (**a**) Mg-5Li-3Al-2Zn-0.2Y; (**b**) Mg-8Li-3Al-2Zn-0.2Y; (**c**) Mg-11Li-3Al-2Zn-0.2Y.

Figure 11 shows the mechanical properties of the three alloys at different conditions. In Mg-5Li-3Al-2Zn-0.2Y (see Figure 11a), during annealing (473 K–573 K), the strength revealed the declining trend with increasing temperature. The ultimate tensile strength decreased from the original value of 274.3 MPa (at room temperature) to 157.8 MPa (at 573 K). In contrast, the elongation increased from 6.7% to 23.8%. This variation should be ascribed to the recrystallization softening effect. In the recrystallization process, the large number of dislocations was absorbed to effectively promote the grain boundary diffusion in which no strengthening factor was generated simultaneously. Furthermore, the reduction of dislocation density also contributed to enhancing the crystal slips. Thereby, the tensile properties of the Mg-5Li-3Al-2Zn-0.2Y alloy exhibited the weakening law during annealing treatment.

In Mg-8Li-3Al-2Zn-0.2Y (see Figure 11b), the tensile properties of the alloy were strengthened by increasing the temperature during annealing. The ultimate tensile strength increased from the original value of 202.2 MPa (at room temperature) to 251.6 MPa (at 573 K). At the same time, the elongation decreased from 26.5% to 16.8%. There existed a similar variation law arising in the tensile properties of the Mg-11Li-3Al-2Zn-0.2Y alloy (see Figure 11c), wherein the ultimate tensile strength increased from the original value of 182.3 MPa (at room temperature) to 265.3 MPa (at 573 K), and simultaneously the elongation decreased from 25.6% to 4.5%.

This phenomenon whereby the annealing process improved the properties of the alloy should be ascribed to the effect of solid solution strengthening. This solid solution mechanism could be measured by the interaction energy between the dissolved atom and dislocation, as shown in Equation (5). Equation (5) could be further simplified as Equation (6).

(5) $$\left\{ \begin{array}{l} E=-p\cdot \Delta V\\ p=\frac{1}{3}\left(\sigma_{xx}+\sigma_{zz}+\sigma_{yy}\right)=-\frac{1}{3}\cdot \frac{1+n}{1-n}\cdot \frac{Gb}{\pi }\cdot \frac{y}{x^{2}+y^{2}}\\ r^{2}=x^{2}+y^{2}\\ \Delta V=4\pi R_{0}^{3}\varepsilon \\ \varepsilon =\frac{R-R_{0}}{R} \end{array}\right.$$

(6) $$E=\frac{4}{3}\cdot \frac{1+n}{1-n}\cdot GbR_{0}^{3}\varepsilon \cdot \frac{\sin\theta }{r}$$

where E is the interaction energy, p is the normal stress on the dislocation, n is the poisson ratio, G is the shear modulus, b is the burgers vector, r is the distance between the point defect and dislocation, ΔV is the lattice volume change, $R_{0}$ is the radius of the matrix atom, R is the radius of the dissolved atom, and sinθ is *y*/*r*. 

As indicated by Equations (5) and (6), the attraction between the dissolved atoms and dislocations will occur when *E* is positive. In contrast, the repulsion between the dissolved atoms and dislocations will occur when *E* is negative. Furthermore, *E* was inversely proportional to *r*, indicating that the smaller the distance between the point defect and dislocation, the higher the value of |*E*|. In addition, the strengthening mechanism can be explained by the dissolved atoms, which had a “pinning” effect on the dislocations. The theory was that the dissolved atoms were segregated around the dislocation line because of the elastic interaction between the dissolved atoms and dislocations. Thereby, the dislocation movements changed the equilibrium position of dissolved atoms, which gave rise to enhancing the system energy and restraining dislocation movements. This restraining extent could be defined by pinning stress (*τ*), as shown in Equations (7) and (8).

(7) $$\left\{ \begin{array}{l} \tau =\frac{f_{\max}}{b}\\ f_{\max}=\frac{3\sqrt{3}A}{8br_{0}^{2}}\\ A=\frac{4}{3}(\frac{1+n}{1-n})GbR_{0}^{3}\varepsilon =const \end{array}\right.$$

(8) $$\tau =\frac{3\sqrt{3}}{8b^{2}r_{0}^{2}}\cdot \frac{4}{3}(\frac{1+n}{1-n})GbR_{0}^{3}\varepsilon$$

where τ is the pinning stress, $f_{\max}$ is the highest force on the dislocation, and $r_{0}$ is the radius of edge dislocation.

### 3.3. Establishing Mathematical Relationship 

To quantitatively measure the strengthening effect, the relationship between the microstructure and mechanical properties should be proposed and determined. Hence, the fitting method with a high accuracy is used to determine their relationship models. The fitting accuracy is measured in terms of the relative error (*RE*), the mean relative error (*MRE*), and the mean square error (*MS*), as shown in Equation (9). The strength and AlLi volume fraction at different temperatures are listed in Table 2.

(9) $$\left\{ \begin{array}{l} RE=\frac{\left|q_{i}-Q_{i}\right|}{Q_{i}}\times 100\%\\ MRE=\frac{1}{n}\sum_{i=1}^{n}\frac{\left|q_{i}-Q_{i}\right|}{Q_{i}}\times 100\%\\ MS=\sqrt{\frac{1}{n}\sum_{i=1}^{n}{\left(q_{i}-Q_{i}\right)}^{2}} \end{array}\right.$$

where $Q_{i}$ is the experimental value, $q_{i}$ is the calculated value from the model, and n is the experimental number.

**Figure 12:**
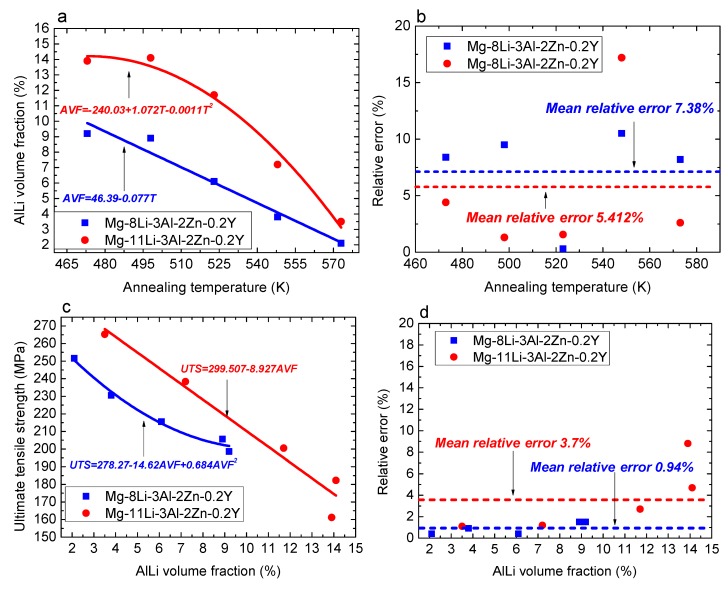
Fitting relationship results: (**a**) AlLi volume fraction on annealing temperature; (**b**) fitting accuracy of Equation (10); (**c**) ultimate tensile strength on AlLi volume fraction; (**d**) fitting accuracy of Equation (11).

Figure 12 shows the fitting relationship results of Mg-8Li-3Al-2Zn-0.2Y and Mg-11Li-3Al-2Zn-0.2Y. As indicated in Figure 12a, based on the evolution characteristic between the AlLi volume fraction and annealing temperature, the fitting method adopted the linear fitting and quadratic polynomial fitting, respectively. The fitting equations of Mg-8Li-3Al-2Zn-0.2Y and Mg-11Li-3Al-2Zn-0.2Y are shown in Equation (10). According to the fitting accuracy (see Figure 12b), the mean relative error for Mg-8Li-3Al-2Zn-0.2Y and Mg-11Li-3Al-2Zn-0.2Y was 7.38% and 5.412%, respectively. The fitting results indicated that the effect of the annealing temperature on the dissolution number of AlLi phase in Mg-8Li-3Al-2Zn-0.2Y was relatively more remarkable than that in Mg-11Li-3Al-2Zn-0.2Y. Based on the analysis, α-Mg matrix occupied a certain amount of area in the Mg-8Li-3Al-2Zn-0.2Y alloy. Furthermore, a large amount of AlLi phase was distributed in the β-Li matrix instead of the α-Mg matrix. Thereby, compared with Mg-11Li-3Al-2Zn-0.2Y, the AlLi phase dissolution in Mg-8Li-3Al-2Zn-0.2Y was more sensitive to the annealing temperature. Figure 12c shows the fitting results between the ultimate tensile strength and AlLi volume fraction, wherein the fitting equations are shown in Equation (11). The corresponding mean relative error for Mg-8Li-3Al-2Zn-0.2Y and Mg-11Li-3Al-2Zn-0.2Y was 0.94% and 3.7%, respectively, as shown in Figure 12d. Compared with the previous fitting results, the mean relative error for Mg-8Li-3Al-2Zn-0.2Y and Mg-11Li-3Al-2Zn-0.2Y decreased by 6.44% and 1.712%, respectively, indicating that the fitting accuracy increased. It also indicated that the relationship between the strength and dissolution number was much closer.

(10) $$AVF=\left\{ \begin{array}{ll} 46.39-0.77T & Mg-8Li-3Al-2Zn-0.2Y\\ -240.03+1.072T-0.0011T^{2} & Mg-11Li-3Al-2Zn-0.2Y \end{array}\right.$$

(11) $$UTS=\left\{ \begin{array}{ll} 278.27-14.62AVF+0.684AVF^{2} & Mg-8Li-3Al-2Zn-0.2Y\\ 299.507-8.927AVF & Mg-11Li-3Al-2Zn-0.2Y \end{array}\right.$$

**Figure 13:**
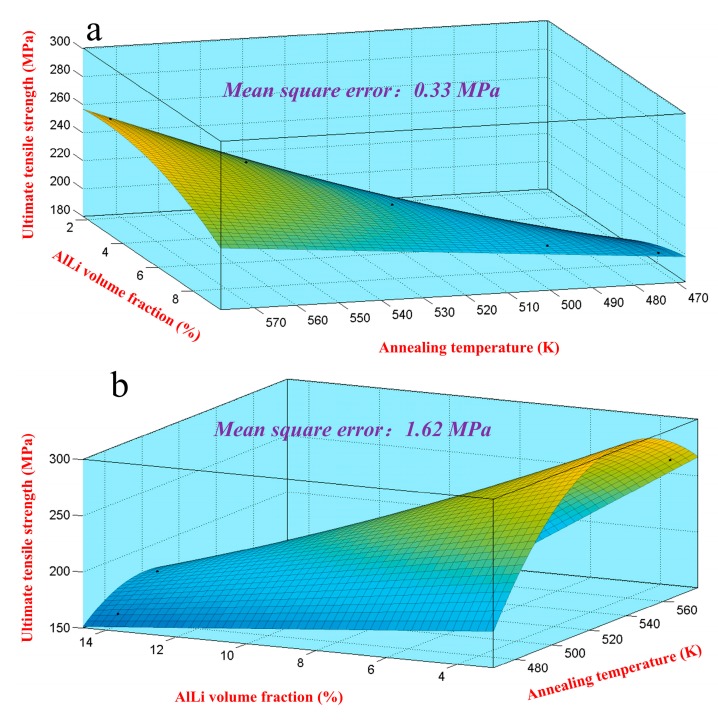
Fitting map of ultimate tensile strength under different volume fractions and temperatures: (**a**) Mg-8Li-3Al-2Zn-0.2Y; (**b**) Mg-11Li-3Al-2Zn-0.2Y.

**Figure 14:**
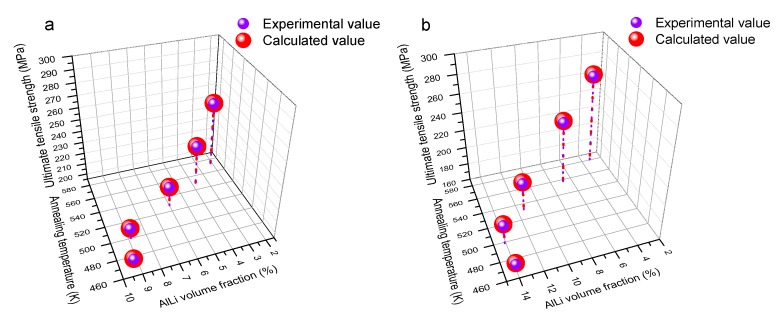
Comparison between experimental value and calculated value of Equation (12): (**a**) Mg-8Li-3Al-2Zn-0.2Y; (**b**) Mg-11Li-3Al-2Zn-0.2Y.

To accurately make a quantitative analysis for the strength on multi-condition, the relationship between the ultimate tensile strength, dissolution number, and annealing temperature should be proposed and determined. The fitting map of ultimate tensile strength under different volume fractions and temperatures is shown in Figure 13. The corresponding fitting equations are shown in Equation (12). Considering the fitting accuracy, the mean square error (*MS*) was calculated by Equation (9). The result manifested that the mean square error for Mg-8Li-3Al-2Zn-0.2Y and Mg-11Li-3Al-2Zn-0.2Y was 0.33 MPa and 1.62 MPa, respectively. Thereby, the fitting accuracy of their relationship models was relatively high. Figure 14 shows the comparison between the experimental value and calculated value of Equation (12). The comparison results also verified the relatively high fitting accuracy on their relationship models.

(12) $$UTS(473K-573K)=\left\{ \begin{array}{ll} -507.3+1.312T+69.68AVF-0.11T\cdot AVF-0.77AVF^{2} & Mg-8Li-3Al-2Zn-0.2Y\\ -6946+26.61T+69.3AVF-0.15T\cdot AVF+0.024T^{2} & Mg-11Li-3Al-2Zn-0.2Y \end{array}\right.$$

## 4. Conclusions

With increasing Li content, an α-Mg matrix was gradually transformed to a β-Li matrix. Meanwhile, the alloy plasticity was obviously enhanced due to decreasing the c/a axis ratio of Mg, as well as activating other non-basal slips. During annealing, the rolled microstructure (high energy zone) in Mg-5Li-3Al-2Zn-0.2Y and Mg-8Li-3Al-2Zn-0.2Y was gradually substituted by the recrystallized microstructure. Furthermore, a kind of abnormal grain growth was observed in Mg-11Li-3Al-2Zn-0.2Y, but not detected in Mg-5Li-3Al-2Zn-0.2Y and Mg-8Li-3Al-2Zn-0.2Y. In addition, a kind of solid solution in the β-Li matrix gradually strengthened the properties of the alloy. To quantitatively analyze this strengthening effect, mathematical modeling was used to determine the relationship between strength and multiple factors.

## Figures and Tables

**Table 1:** Crystal structure parameters of Al_2_Y.

Space Group	Atom Number in Primitive Cell	Atom Site	Pearson Sign	Equilibrium Crystal Parameters (nm)	Unit Cell Volume (nm^3^)	Density (g/cm^3^)
Fd 3m (227)	6	Al: (0.625,0.625,0.625) Y: (0,0,0)	cF24	0.554	0.124	3.838

**Table 2:** Strength and AlLi volume fraction at different temperatures.

Designed Alloy	Annealing Temperature (K)/T	AlLi Volume Fraction (%)/AVF	Ultimate Tensile Stress (MPa)/UTS
Mg-8Li-3Al-2Zn-0.2Y	473 K	9.2	198.6
	498 K	8.9	205.6
	523 K	6.1	215.6
	548 K	3.8	230.5
	573 K	2.1	251.6
Mg-11Li-3Al-2Zn-0.2Y	473 K	13.9	161.2
	498 K	14.1	182.3
	523 K	11.7	200.5
	548 K	7.2	238.3
	573 K	3.5	265.3
